# A randomized clinical trial on the effectiveness of an intervention to treat psychological distress and improve quality of life after autologous stem cell transplantation

**DOI:** 10.1007/s00277-015-2509-6

**Published:** 2015-09-30

**Authors:** Annemarie M. J. Braamse, B. van Meijel, O. J. Visser, A. D. Boenink, P. Cuijpers, C. E. Eeltink, A. W. Hoogendoorn, M. van Marwijk Kooy, P. van Oppen, P. C. Huijgens, A. T. F. Beekman, J. Dekker

**Affiliations:** Department of Psychiatry and EMGO Institute for Health and Care Research, VU University Medical Center, A.J.Ernststraat 1187, Amsterdam, 1081 HL Netherlands; Department of Health, Sports & Welfare/Cluster Nursing, Inholland University of Applied Sciences, Amsterdam, Netherlands; Parnassia Psychiatric Institute, The Hague, Netherlands; Department of Hematology, VU University Medical Center, Amsterdam, Netherlands; Department of Clinical Psychology, VU University, Amsterdam, Netherlands; Department of Oncology/Hematology, Isala Clinics, Zwolle, Netherlands; Department of Rehabilitation Medicine, VU University Medical Center, Amsterdam, Netherlands

**Keywords:** Depression, Anxiety, Quality of life, Hematological neoplasms, Hematopoietic stem cell transplantation

## Abstract

Psychological distress contributes to impaired quality of life in hematological cancer patients. Stepped care treatment, in which patients start with the least intensive treatment most likely to work and only receive more intensive interventions if needed, could improve distress. We aimed to evaluate the outcome of stepped care treatment on psychological distress and physical functioning in patients treated with autologous stem cell transplantation for hematological malignancies. In the present study, we performed a randomized clinical trial with two treatment arms: stepped care and care as usual. Baseline assessment and randomization occurred during pre-transplant hospitalization. Stepped care was initiated after 6 weeks, consisting of (1) watchful waiting, (2) Internet-based self-help intervention, and (3) face-to-face counseling/ psychopharmacological treatment/ referral. Follow-up measurements were conducted at 13, 30, and 42 weeks after transplantation. Stepped care (*n* = 47) and care as usual (*n* = 48) were comparable on baseline characteristics. The uptake of the intervention was low: 24 patients started with step 1, 23 with step 2, and none with step 3. Percentages of distressed patients ranged from 4.1 to 9.7 %. Ten percent of patients received external psychological or psychiatric care. No statistically significant differences were found between stepped care and care as usual on psychological distress or physical functioning in intention to treat analyses, nor in per protocol analyses. The stepped care program was not effective in decreasing psychological distress. The low intervention uptake, probably related to the low levels of psychological distress, offers an explanation for this outcome. Future research should take into account patients’ specific care needs.

*Netherlands Trial Registry identifier*: NTR1770.

## Background

For patients diagnosed with hematological malignancies, autologous stem cell transplantation (auto-SCT) following high-dose chemotherapy is a common treatment option. Treatment with auto-SCT generally leads to improved survival, with an overall 60 % probability of surviving 5 years from transplantation [1]. At the same time, auto-SCT survivors are confronted with impairments in their health-related quality of life (QOL) [2, 3]. Before and shortly after auto-SCT, patients generally face impairments in physical, emotional, and role functioning. Although most patients return to or surpass pre-transplant levels of functioning in subsequent months and years, continuous impairments are observed in physical functioning, role functioning, and global QOL [2, 3].

A strong predictor of QOL after auto-SCT is the presence of psychological distress [4, 5]. Psychological distress is a multifactorial concept, encompassing common feelings of vulnerability, sadness, and fears, as well as potentially disabling problems such as depression, anxiety, or social isolation [6]. In the present study, psychological distress is defined as the presence of elevated depressive or anxiety symptoms. Previous research shows large variation in the prevalence of depressive and anxiety symptoms in the period from pre-transplant to 1 year post-transplant. Prevalence rates of 5 to 48 % have been reported for elevated depressive symptoms, and 5 to 45 % for anxiety symptoms [4, 7–10]; the period of initial hospitalization has been pointed at as being most stressful [10, 11]. Patients with elevated depressive or anxiety symptoms before or during transplantation more often face impaired post-transplant psychological functioning [4, 5, 12, 13] and physical limitations [4].

Problem Solving Treatment is an effective intervention for reducing psychological distress [14], also in cancer survivors [15]. It aims at strengthening patients’ self-management skills to solve present and future problems. Patients learn to (a) prioritize problems which matter most to them and which in principle can be solved, (b) analyze the problem and generate alternative solutions, (c) select methods for solution and implement these, and (d) evaluate the results and prepare for the future. This may help patients cope with the challenges they encounter related to disease and treatment. Since psychological distress seems to contribute to impaired QOL after auto-SCT, successful treatment of psychological distress is expected to improve patients’ QOL. In delivering treatment for psychological distress, the stepped care approach has been strongly advocated [16]. In this approach, patients start with the least intensive treatment that is most likely to work. Only those patients insufficiently helped by the initial treatment receive more intensive and costly interventions. Stepped care aims at an effective and efficient allocation of therapeutic resources.

In the present study, psychological support, organized according to a stepped care approach, was offered to patients treated with auto-SCT for hematological malignancies. The steps included (1) watchful waiting; (2) Internet-based self-help program, based on the principles of Problem Solving Treatment; and (3) individual face-to-face counseling, medication, or referral to other services. We aimed to evaluate the outcome of stepped care on psychological distress and the QOL domain physical functioning. It was hypothesized that stepped care would result in improvement of psychological distress, and thereby in improvement of physical functioning.

## Methods

### Design

In this pragmatic two-armed randomized clinical trial, stepped care was compared with care as usual. An extensive description of the study protocol has been published previously [17]. The study was approved by the Medical Ethics Committees of the participating hospitals: VU University Medical Center, Amsterdam, and Isala Clinics, Zwolle. All procedures followed were in accordance with the ethical standards of the Medical Ethics Committees and with the Helsinki Declaration of 1975, as revised in 2008. The trial was registered in the Netherlands Trial Registry (NTR1770).

### Participants and setting

Patients were recruited from the hematology departments at the two participating hospitals in the Netherlands between August 2009 and April 2013. Eligibility criteria comprised a diagnosis of hematological malignancy (multiple myeloma, (non-)Hodgkin lymphoma, or acute leukemia), scheduled for treatment with high-dose chemotherapy and auto-SCT; age ≥18 years; and life expectation >3 months. Exclusion criteria comprised an insufficient command of the Dutch language, and a contraindication for the stepped care approach (e.g., hospitalization in a mental health institute). All participants provided written informed consent.

The majority of patients received follow-up care after auto-SCT at the hospital where they had been transplanted. However, 41 % of the study participants were redirected to their local hospital for follow-up care.

### Procedures

**Fig. 1:**
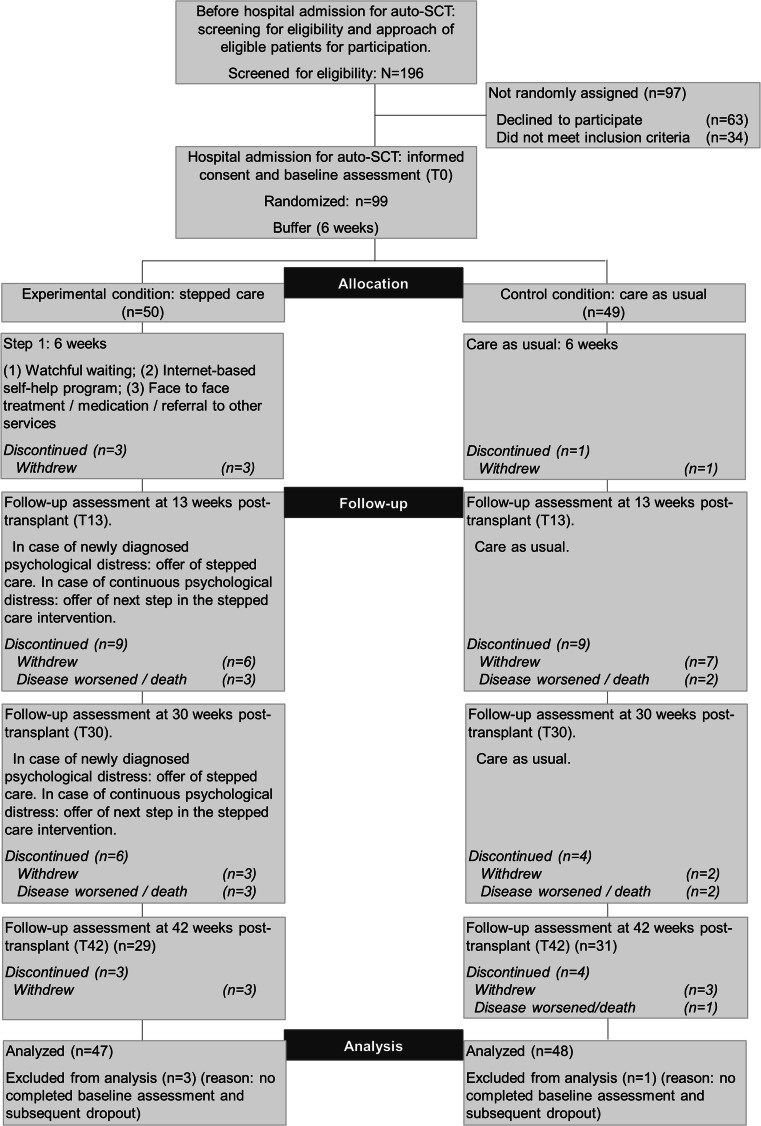
Design of the randomized clinical trial/CONSORT flow diagram

At hospital admission for high-dose chemotherapy and auto-SCT, patients who provided informed consent filled in the baseline questionnaire (T0). Subsequently, patients were allocated at random to one of the two study arms: stepped care or care as usual. Stepped care was initiated after a 6-week buffer period, allowing for initial physical recovery. Follow-up measurements were administered by mail at 13 weeks (T13), 30 weeks (T30), and 42 weeks (T42) after transplantation. The design is illustrated in Fig. 1.

Randomization was performed by the second author, who was not involved in data collection and analysis, using a random digit generator. Randomization was stratified for treating hospital (VU University Medical Center vs Isala Clinics) and diagnosis (multiple myeloma vs lymphoma). Patients were informed about their assignment by the first author during the 6-week buffer period. Due to the nature of the intervention, neither patients nor health care providers could be blinded to the intervention. Randomization and statistical analysis were performed blindly.

### Interventions

Under the assumption that all patients could benefit from improved problem-solving skills, all patients in the experimental study arm were, regardless of whether psychological distress was present, provided with a stepped care program which contained three steps: watchful waiting, an Internet-based self-help intervention, and individual face-to-face counseling/psychopharmacological treatment/referral to other services (see below). At time of the randomization assignment, patients were asked with which step they wanted to start. Step 1 (watchful waiting) was the default choice, but patients were allowed to start with step 2 or 3 if they wished to. At 13- and 30-week follow-up, patients with elevated psychological distress were contacted and asked if they wanted to progress to a next step in the stepped care program.

#### Step 1: Watchful waiting

Since psychological distress often improves without requiring active intervention, the first step consisted of 6 weeks of watchful waiting. This step was added to the study protocol [17], as we noticed during the study that there was a need for a less intensive program option: For some patients, the Internet-based self-help intervention appeared to be too intensive as the default first step of the program. Therefore, we made the program more broadly accessible by adding watchful waiting. At that point in time, 30 patients had already been included in the study, of which 15 patients had been randomly allocated to stepped care. Before adding this step to the protocol, step 2 (Internet-based self-help intervention) was the default choice.

#### Step 2: Internet-based self-help intervention

Patients were provided with an Internet-based self-help intervention, based on the principles of Problem Solving Treatment. Previous studies confirmed the effectiveness of this intervention in treating psychological distress [18, 19]. The original intervention, called Everything Under Control [18], was adapted to our patient group [17] in close collaboration with nurses, nurse practitioners, and hematologist-oncologists from the Department of Hematology: Specific information on disease and treatment was added, and texts and examples were adjusted to make sure these applied to auto-SCT patients. The intervention consisted of five modules with information, examples, and exercises. Patients were asked to work through one module a week, with the advice to spend approximately 2 h per module, and send their completed assignments to their coach. Support was provided by a trained psychologist and consisted of brief, weekly e-mails in reply to patients’ assignments and a weekly standardized e-mail to announce a new module. Support was merely intended to facilitate patients’ effective use of the intervention. Patients could contact their coach at any moment for additional support via e-mail or the website. A small pilot test (*n* = 3) was carried out before the start of the study to ensure applicability and feasibility of the intervention. For patients without access to the Internet, the intervention was available in booklet. The content of the booklet was exactly similar to the website. In these cases, coaching was arranged via telephone or e-mail.

#### Step 3: Individual face-to-face counseling, psychopharmacological treatment, or referral to other services

For patients in step 3, a collaborative care team consisting of a consultant psychiatrist, psychiatric nurse consultant, nurse practitioner (Department of Hematology), hematologist-oncologist, and patient was formed. This team, coordinated by the nurse practitioner, evaluated the patient’s need for treatment and developed a treatment plan. Diagnostic evaluation was performed by the consultant psychiatrist, psychiatric nurse consultant, and nurse practitioner, using the following instruments: the anxiety and depression modules of the Composite International Diagnostic Interview (CIDI), version 2.1 [20], Camberwell Assessment of Need (CAN) [21], and Intermed [22]. Subsequently, the psychiatric nurse consultant discussed the following treatment options with the patient: individual face-to-face counseling based on the principles of Problem Solving Treatment, psychopharmacological treatment, and referral to other health care or social services.

In the control condition of the study, patients were provided with *care as usual*. Care as usual represented regular care at the Department of Hematology in the two hospitals. During regular follow-up visits, emotional support was provided by the hematologist-oncologists and nurses on an ad hoc basis. If patients brought up any problem, hematologist-oncologists or nurses could undertake action and/or refer patients to other services.

### Measures

The following validated and reliable self-report questionnaires were included in the assessment battery: Hospital Anxiety and Depression Scale (HADS) [23], European Organization for Research and Treatment of Cancer Quality of Life Questionnaire-C30 (EORTC QLQ-C30) [24] version 3.0, Patient Health Questionnaire-9 (PHQ-9) [25], Spielberger State-Trait Anxiety Inventory: state version (STAI-state) [26, 27], Social Problem Solving Inventory-Revised (SPSI-R) [28], and the Dutch General Self-efficacy Scale (DGSS) [29]. A checklist, specifically developed for this trial, was used to collect data on additional supportive care patients received. Demographic information was collected at baseline, and medical data were extracted from the patient’s medical record. To determine whether elevated psychological distress was present, we used the HADS (cutoff ≥8 on one or both of the subscales, or ≥15 on the overall questionnaire), PHQ-9 (cutoff ≥10), and STAI-state (cutoff ≥40).

The primary outcomes of the current study were psychological distress (anxiety and depression) (HADS), and physical functioning (EORTC QLQ-C30). Secondary outcomes included emotional functioning and role functioning (EORTC QLQ-C30), other measures of psychological distress (STAI, PHQ), problem solving (SPSI-R), and self-efficacy (DGSS).

### Data analysis

Descriptive statistics were used to compare baseline characteristics of the experimental and control group, and to compare baseline characteristics of dropouts and completers in the total sample. Intention-to-treat and per-protocol analyses were conducted. For the intention to treat analyses, linear mixed-model analyses were performed to evaluate the difference in outcome (psychological distress, physical functioning, and secondary outcomes) between the experimental group and the control group. A random intercept was used, and condition and time were fixed effects. A group * time interaction was added to the model to test for treatment effects over time. Based on pooled pre-test standard deviations [30], effect sizes were calculated for the estimated differences between T0 and T13, T0 and T30, and T0 and T42, between groups. To test whether baseline level of distress moderated the effect of the intervention in the two groups, a three-way interaction term (baseline distress * group * time) was added to the model. Since linear mixed-model analysis is able to handle missing observations due to dropout, no additional actions were undertaken for handling missing data.

Two per-protocol analyses were carried out, following the same procedure as in the intention-to-treat analyses. In the first per-protocol analysis, we excluded patients in the experimental group if (a) despite presence of elevated distress after completing watchful waiting or the Internet-based self-help program, they did not enter a next step of the program; or (b) they followed less than four lessons of the Internet-based self-help intervention. In the second analysis, we only included patients who had completed at least four lessons of the Internet-based self-help program. Patients who had received psychological or psychiatric care outside of the study were excluded from both per protocol analyses. Data were analyzed using IBM SPSS statistics version 20.0 (IBM SPSS Statistics for Windows, Armonk, NY).

### Sample size

The power calculation concerned the comparison at T30 compared to T0 between the two groups (stepped care vs. care as usual). A meta-analysis on Problem Solving Therapy for mental and physical health problems has documented an effect size of *d* = 0.54, compared to treatment as usual [14]. Setting *d* = 0.5, alpha = 0.05 (two tail), and beta = 0.80, the required sample size was 2 × 64 = 128 patients. A second power calculation was made, considering the difference in treatment effect over time (T0, T13, T30, and T42). Setting the within-subject correlation coefficient (rho) at 0.5, the required sample size was 2 × 42 = 84 patients.

## Results

Of 162 eligible patients, 99 (response rate 61.1 %) agreed to participate, provided informed consent, filled in the baseline questionnaire (T0), and were randomly assigned to one of the two treatment arms. The main reason for non-participation was considering participation to take too much energy or effort. Some patients refused study participation in general, and a few patients had no interest in psychosocial research. After randomization, four patients appeared to have completed the baseline questionnaire only partly, and they declined further participation. Data of these patients was therefore not analyzed. Of the remaining 95 patients, 78 % (T13), 66 % (T30), and 63 % (T42) completed the follow-up assessments (Fig. 1).

Table 1 presents the baseline demographic and clinical characteristics of our sample. Patients in the two study arms were comparable. When comparing baseline characteristics of patients who completed the study and patients who dropped out before the last assessment, completers were significantly older and lived with a partner, had multiple myeloma (as opposed to lymphoma), and received high-dose melphalan (HDM) as conditioning regimen more often (all *P* < 0.05).

**Table 1:** Sample characteristics

	Stepped care (*n* = 47)	Care as usual (*n* = 48)	*P* value
Gender, female, *n* (%)	16 (34.0)	14 (29.2)	0.61
Age in years, mean (s.d.)	53.3 (8.7)	55.5 (8.7)	0.23
BMI, mean (s.d.)	25.8 (4.0)	26.4 (3.2)	0.41
Living with partner, yes, *n* (%)^a^	33 (70.2)	40 (85.1)	0.08
Education, *n* (%)^a^			0.06
<4-year college degree	16 (34.0)	25 (53.2)	
≥4-year college degree	31 (66.0)	22 (46.8)	
Diagnosis, *n* (%)			0.77
Non-Hodgkin lymphoma	19 (40.4)	17 (35.4)	
Hodgkin lymphoma	5 (10.6)	4 (8.3)	
Multiple myeloma	23 (48.9)	27 (56.3)	
Type of conditioning, *n* (%)			0.36
High-dose melphalan (HDM)	23 (48.9)	28 (58.3)	
BEAM/Z-BEAM	24 (51.1)	19 (39.6)	
Busulfan/cyclofosfamide	–	1 (2.1)	
Somatic comorbidities, *n* (%)^a^			0.39
None	6 (12.8)	2 (4.3)	
1	19 (40.4)	18 (38.3)	
2	8 (17.0)	12 (25.5)	
3 or more	14 (29.8)	15 (31.9)	
Remission status at time of transplant^b^			0.85
Complete remission	19 (44.2)	19 (42.2)	
Not in complete remission	24 (55.8)	26 (57.8)	

^a^Care as usual: 1 patient missing data

^b^Stepped care: 4 patients missing data; care as usual: 3 patients missing data

Overall, external psychological or psychiatric care (independent of the study) was received by 10.3 % of patients during the study period (5.9 % at T13, 5.7 % at T30, and 7.4 % at T42).

Of the 47 patients who started with the stepped care program, 24 patients started with step 1 (watchful waiting); 1 patient subsequently entered step 2 because of psychological distress. Twenty-three patients started with step 2 (Internet-based self-help intervention), of which 15 entered this step by default, before watchful waiting was added to the program. None of the patients entered step 3 (individual face-to-face counseling, psychopharmacological treatment, or referral). Except for one patient, all patients followed only one step of the stepped care program.

In total, 24 patients took the Internet-based self-help intervention. Ten patients completed the intervention (at least four out of five lessons). Reasons for dropout (*n* = 14) were feeling capable of coping by themselves (nine patients); not wanting to deal with psychosocial aspects of disease and treatment at this point in time (three patients); being too ill to continue (one patient); and feeling that one’s problems could not be addressed well enough in a self-help intervention, but not wanting to travel to our hospital for face-to-face counseling (one patient). Dropout was independent of the presence of psychological distress: Of the eight patients with psychological distress who entered step 2, 3 completed the intervention; of the 16 patients without psychological distress, 8 completed the intervention.

In both study arms, HADS total scores were relatively low on all four measurement points. However, the large standard deviations in our sample point to substantial individual differences. Percentages of patients scoring ≥15 ranged from 4.1 to 9.7 %. Percentages of distressed patients on the HADS subscales and other questionnaires are presented in Table 2.

**Table 2:** Percentages of patients with psychological distress

		T0	T13	T30	T42
		Total *n* = 95 SC *n* = 47 CAU *n* = 48	Total *n* = 77 SC *n* = 38 CAU *n* = 39	Total *n* = 67 SC *n* = 32 CAU *n* = 35	Total *n* = 60 SC *n* = 29 CAU *n* = 31
Psychological distress (HADS total score, cutoff ≥15)	Total group	7.5	4.1	9.7	8.5
	Stepped care	4.3	8.3	13.3	14.3
	Care as usual	10.9	0	6.3	3.2
Depression (HADS subscale, cutoff ≥8)	Total group	7.5	9.6	11.3	10.2
	Stepped care	2.1	11.1	10.0	10.7
	Care as usual	13.0	8.1	12.5	9.7
Anxiety (HADS subscale, cutoff ≥8)	Total group	6.5	8.2	12.9	11.9
	Stepped care	6.4	8.3	16.7	17.9
	Care as usual	6.5	8.1	9.4	6.5
Depression (PHQ, cutoff ≥10)	Total group	16.0	5.4	12.9	8.5
	Stepped care	12.8	8.3	13.3	10.7
	Care as usual	19.1	2.6	12.5	6.5
Anxiety (STAI, cutoff ≥40)	Total group	27.7	19.2	19.4	20.3
	Stepped care	23.4	22.2	16.7	14.3
	Care as usual	31.9	16.2	21.9	25.8

*SC* stepped care, *CAU* care as usual, *HADS* Hospital Anxiety and Depression Scale, *PHQ* Patient Health Questionnaire, *STAI* Spielberger State-Trait Anxiety Inventory

The results of the intention to treat analyses are summarized in Tables 3 and 4. Table 3 presents the observed means and standard deviations of the HADS total scores, EORTC physical functioning (primary outcomes), and secondary outcomes. Table 4 summarizes the results of the linear mixed models analyses. Comparing stepped care and care as usual on the HADS total scores and EORTC physical functioning, no statistically significant differences were found. Baseline level of distress did not moderate the effect of the intervention on distress, nor on physical functioning, in the two groups (*P* > 0.10 at T13, T30, and T42; data not shown).

**Table 3:** Observed mean scores of primary and secondary outcomes

		Stepped care *N* = 47	Care as usual *N* = 48
		Mean (s.d.)	Mean (s.d.)
*Primary outcomes*			
Psychological distress (HADS total score)	T0	6.72 (4.47)	7.26 (4.90)
	T13	6.69 (4.96)	5.75 (3.45)
	T30	6.53 (6.06)	6.38 (5.93)
	T42	6.54 (6.44)	6.29 (4.55)
Depression (HADS subscale)	T0	2.98 (2.24)	3.52 (3.38)
	T13	3.17 (2.55)	2.73 (2.51)
	T30	2.63 (2.91)	3.13 (4.03)
	T42	2.64 (2.88)	2.87 (3.03)
Anxiety (HADS subscale)	T0	3.74 (2.88)	3.74 (2.64)
	T13	3.53 (2.89)	3.03 (2.21)
	T30	3.90 (3.58)	3.25 (2.75)
	T42	3.89 (3.93)	3.42 (2.43)
Physical functioning (EORTC QLQ-C30)	T0	74.47 (20.06)	70.64 (19.59)
	T13	74.25 (21.91)	74.05 (19.55)
	T30	82.22 (17.00)	79.79 (23.74)
	T42	83.10 (15.76)	82.80 (18.28)
*Secondary outcomes*			
Emotional functioning (EORTC QLQ-C30)	T0	80.50 (17.83)	79.89 (16.16)
	T13	82.41 (16.64)	87.84 (13.55)
	T30	84.17 (17.28)	84.38 (14.93)
	T42	83.93 (17.85)	81.18 (14.59)
Role functioning (EORTC QLQ-C30)	T0	59.22 (35.24)	57.09 (34.18)
	T13	68.06 (28.56)	66.67 (24.46)
	T30	75.00 (27.60)	75.52 (29.63)
	T42	74.40 (27.02)	74.19 (27.50)
Anxiety (STAI)	T0	35.06 (8.74)	35.15 (8.18)
	T13	32.97 (8.76)	31.57 (7.90)
	T30	32.60 (10.22)	33.44 (10.33)
	T42	32.89 (11.93)	33.10 (9.69)
Depression (PHQ)	T0	5.66 (4.07)	4.87 (4.96)
	T13	4.61 (3.72)	3.82 (2.65)
	T30	3.93 (4.16)	4.84 (5.73)
	T42	4.14 (3.66)	3.68 (3.45)
Problem solving (SPSI-R)	T0	133.36 (19.86)	137.34 (18.36)
	T13	138.86 (12.43)	135.56 (20.92)
	T30	137.48 (14.11)	139.25 (17.29)
	T42	140.41 (17.46)	136.63 (18.58)
Self-efficacy (DGSS)	T0	31.79 (3.92)	32.20 (5.28)
	T13	33.28 (4.42)	32.38 (4.95)
	T30	33.17 (4.15)	31.94 (5.10)
	T42	33.07 (4.95)	31.57 (6.04)

*HADS* Hospital Anxiety and Depression Scale, *EORTC QLQ-C30* European Organization for Research and Treatment of Cancer Quality of Life Questionnaire-C30, *SPSI-R* Social Problem Solving Inventory-Revised, *DGSS* Dutch General Self-efficacy Scale, *PHQ* Patient Health Questionnaire, *STAI* Spielberger State-Trait Anxiety Inventory

**Table 4:** Test statistics and effect sizes of the differences in primary and secondary outcomes between the experimental group and the control group, from linear mixed model analyses

		*t*	*P*	Effect size
*Primary outcomes*				
Psychological distress (HADS total score)	Condition*T13	1.40	0.16	0.28
	Condition*T30	0.47	0.64	0.10
	Condition*T42	0.50	0.62	0.11
Depression (HADS subscale)	Condition*T13	1.31	0.19	0.30
	Condition*T30	−0.10	0.92	−0.02
	Condition*T42	0.05	0.96	0.01
Anxiety (HADS subscale)	Condition*T13	0.90	0.37	0.18
	Condition*T30	0.97	0.33	0.21
	Condition*T42	0.87	0.39	0.19
Physical functioning (EORTC QLQ-C30)	Condition*T13	−1.00	0.32	−0.19
	Condition*T30	−0.53	0.60	−0.11
	Condition*T42	−0.12	0.91	−0.02
*Secondary outcomes*				
Emotional functioning (EORTC QLQ-C30)	Condition*T13	−2.32	0.02*	−0.43
	Condition*T30	−0.94	0.35	−0.19
	Condition*T42	0.12	0.91	0.02
Role functioning (EORTC QLQ-C30)	Condition*T13	−0.09	0.93	−0.02
	Condition*T30	−0.52	0.60	−0.11
	Condition*T42	0.15	0.89	0.03
Anxiety (STAI)	Condition*T13	0.99	0.33	0.21
	Condition*T30	−0.29	0.77	−0.07
	Condition*T42	0.16	0.87	0.04
Depression (PHQ)	Condition*T13	0.09	0.93	0.02
	Condition*T30	−1.32	0.19	−0.31
	Condition*T42	−0.54	0.59	−0.13
Problem-solving (SPSI-R)	Condition*T13	1.59	0.11	0.29
	Condition*T30	1.02	0.31	0.20
	Condition*T42	1.61	0.11	0.32
Self-efficacy (DGSS)	Condition*T13	0.99	0.33	0.19
	Condition*T30	1.27	0.21	0.26
	Condition*T42	1.40	0.16	0.29

*HADS* Hospital Anxiety and Depression Scale, *EORTC QLQ-C30* European Organization for Research and Treatment of Cancer Quality of Life Questionnaire-C30, *SPSI-R* Social Problem Solving Inventory-Revised, *DGSS* Dutch General Self-efficacy Scale, *PHQ* Patient Health Questionnaire, *STAI* Spielberger State-Trait Anxiety Inventory

*P < 0.05

On the following secondary outcomes, no statistically significant differences were found between stepped care and care as usual: STAI anxiety, EORTC emotional functioning, EORTC role functioning, PHQ depression, SPSI-R problem solving, and DGSS self-efficacy (except for a significant difference on EORTC emotional functioning at 13 weeks, in favor of the care as usual group (*t* = −2.32, *P* = 0.02, *d* = −0.43)) (Table 4).

The per-protocol analyses yielded similar results as the intention-to-treat analyses, except for the difference on EORTC emotional functioning at 13 weeks: This finding was non-significant in the per-protocol analysis which included only patients who had completed at least four lessons of the Internet-based self-help program.

## Discussion

The purpose of the present study was to evaluate a stepped care program aimed at treating psychological distress and improving QOL in patients with hematological malignancies treated with auto-SCT. The program as presented here was not effective in treating psychological distress in this patient group. The uptake of the program appeared to be very limited.

Few randomized controlled trials have previously evaluated treatment for psychological distress in hematological cancer patients. An Internet-based program for coping with cancer found an effect on fighting spirit, but not on psychological distress [31]. Another intervention was successful in decreasing anxiety during hospitalization [32]. More broadly, in cancer patients, many trials have focused on treating psychological distress. These trials differ substantially with regard to study designs and studied treatments, but in general, small-to-medium effect sizes have been reached [33]. Reflecting on our trial, the response rate of 61.1 % was relatively high. However, the uptake of our intervention was low, which limited the possibility of finding an intervention effect: Of the 47 patients in the experimental condition, only 24 started with the Internet-based self-help intervention. More intensive treatment options (step 3 of the program) were not chosen.

Several factors may explain the low uptake of the intervention program. The first explanation concerns the prevalence of psychological distress. Prevalence rates of elevated anxiety and depressive symptoms at baseline were 6.5 and 7.5 %, respectively; these are equal to some, but lower than other rates found in observational studies [4, 7–10]. Mean distress scores in our sample were lower than in a Dutch population sample of persons aged 18–65 years [23]. The low scores and prevalence rates probably indicate a low need for psychological care in our patient group and/or a high standard of usual care, as 10.3 % of our study participants received psychological or psychiatric care outside of the study program. Together, the low uptake of the intervention, low distress scores, and high standard of usual care probably led to a reduced contrast between the experimental group and the control group.

Given patients’ life-threatening illness and stressful treatment, we assumed beforehand that improved problem-solving skills could help to decrease psychological distress and improve QOL in all patients. We therefore offered the stepped care program to all patients in the experimental study arm, regardless of their level of psychological distress. This may have limited our potential to show an intervention effect: In recent years, it has become clear that patients with elevated levels of distress are the patients most likely to benefit from psychological interventions [33, 34]. Trials showing effective treatment results either only included patients with psychological distress (e.g., [35, 36]) or recruited patients via advertisements [31] and thereby pre-selected study participants with a higher need for and interest in an intervention aimed at psychological distress. However, in our trial, subanalyses with only distressed patients did not show a significant intervention effect. It is suggested that future interventions should target specific care needs, instead of being offered to all patients. In this respect, it is important to note that presence of psychological distress and need for support not necessarily coincide [37]. Besides, whether psychological distress is the best indicator for guiding supportive care is questionable. Targeting care needs could be considered instead [38], in which case a focus on physical and cognitive-emotional needs could be desirable [39]. In addition, cost-effectiveness is an important aspect when implementing interventions and should be evaluated in future studies.

A second factor influencing the uptake of our intervention program could be the nature of the intervention itself. The Internet-based self-help intervention, which was the second step of the program, has previously been shown to be effective [18]. Our dropout rates were relatively high, however. When evaluating the reasons for dropout, the intensity of the program appeared to be too high for those patients who felt capable of coping with their problems themselves. For other patients, the timing of offering the intervention did not match their needs. The third and most intensive step of the program, consisting of face-to-face treatment or other care options, was not chosen by any patient. One interpretation of our results is that Problem Solving Treatment as specific psychotherapeutic technique, as well as the opportunity to receive face-to-face treatment, may not match the needs of the majority of patients. Most hematological cancer patients seem resilient when it comes to coping with their disease and QOL impairments. If needed, other forms of psychological support could be more suitable, such as supporting self-management, or guided peer support, reserving psychotherapeutic care options for those patients with an anxiety or depressive disorder.

Other factors that may have influenced the uptake of the intervention are current health care provision, the timing of offering treatment, and logistic issues. First, during the study period, 10.3 % of the study participants received psychological or psychiatric care outside of the study. This could reflect the relatively high accessibility of additional supportive care in the Dutch health care system. Second, in our stepped care program, providing patients with psychological care options at 6 weeks after transplantation may not have been appropriate. Emotional problems might occur at that point in time, but also in an earlier stage of the disease or treatment process or, conversely, in a later stage. It has been proposed that emotional concerns may only come up after acute physical problems have been dealt with [40]. The timing of offering treatment should be flexible, fitting individual patients’ needs. Finally, the treatment location in step 3 of the stepped care program may have led to logistic barriers. This step was organized in the hospitals where patients had received their auto-SCT. However, about 40 % of the patients were redirected to their local hospital for follow-up care after auto-SCT.

Concluding, the stepped care program as presented in the current study was not effective in decreasing psychological distress. This could mainly be explained by the low uptake of the intervention, probably because of the low baseline levels of psychological distress. Also, whether psychological distress is the best indicator for guiding supportive care is questionable. Future research could look into treatments that target patients’ care needs, and into other ways of support than psychotherapeutic treatment.
